# Effector and Regulatory T Cell Trafficking in Corneal Allograft Rejection

**DOI:** 10.1155/2017/8670280

**Published:** 2017-04-28

**Authors:** Afsaneh Amouzegar, Sunil K. Chauhan

**Affiliations:** ^1^Schepens Eye Research Institute, Massachusetts Eye and Ear Infirmary, Boston, MA, USA; ^2^Department of Ophthalmology, Harvard Medical School, Boston, MA, USA

## Abstract

Corneal transplantation is among the most prevalent and successful forms of solid tissue transplantation in humans. Failure of corneal allograft is mainly due to immune-mediated destruction of the graft, a complex and highly coordinated process that involves elaborate interactions between cells of innate and adaptive immunity. The migration of immune cells to regional lymphoid tissues and to the site of graft plays a central role in the immunopathogenesis of graft rejection. Intricate interactions between adhesion molecules and their counter receptors on immune cells in conjunction with tissue-specific chemokines guide the trafficking of these cells to the draining lymph nodes and ultimately to the site of graft. In this review, we discuss the cascade of chemokines and adhesion molecules that mediate the trafficking of effector and regulatory T cells during corneal allograft rejection.

## 1. Introduction

Corneal transplantation is one of the most successful forms of human solid organ transplantation. With more than 400,000 surgeries performed annually in the US and 100,000 worldwide, corneal transplantation is among the most widely performed transplant procedures in the world [1]. Despite the relatively high-acceptance rates of corneal allografts compared to other tissues, the fate of the corneal graft is highly dependent on the graft bed microenvironment. While the survival rates in normal recipients are approximately 90%, conditions such as host bed inflammation and vascularization or history of previous rejection, which render the host as high-risk, predispose graft recipients to high-failure rates around 50% [2–4].

The development of new surgical techniques and immunosuppressive drugs has considerably increased the success rate of corneal transplants. However, immune-mediated rejection remains the most common cause of graft failure. Although the cornea is an immune-privileged tissue, factors such as inflammation and neovascularization can disrupt this privilege and lead to the development of a graft-directed immune response [5]. The orchestrated response of innate and adaptive immune cells against the alloantigen is highly dependent on directed migration and homing of immune cells to the lymphoid tissues and site of inflammation [6]. This immune cell trafficking is regulated by a complex interplay between adhesion molecules and chemokines and their counter receptors. In this review, we focus on the migration and homing of the two most critical T cell subsets that are involved in graft alloimmunity, effector T cells and regulatory T cells, with an emphasis on the chemokines and adhesion molecules involved in the migration of these cells to the draining lymphoid tissues and the graft site.

## 2. Pathophysiology of Corneal Graft Rejection

Corneal allograft rejection is a multifaceted process that involves complex interactions between cells of innate and adaptive immunity. Response to allogeneic tissue begins following transplantation with upregulation of proinflammatory cytokines, adhesion molecules, and chemokines, which result in mobilization of antigen-presenting cells (APCs) from the vascular compartment and peripheral cornea to the central cornea [7]. These mobilized APCs undergo a maturation process during which they acquire MHC class II and costimulatory molecules such as CD80 and CD86; a phenotypic change that makes them more potent in presenting the alloantigen to T cells [8, 9]. Furthermore, the resultant inflammatory milieu nullifies the effect of antiangiogenic factors, such as PEDF, TSP-1, endostatin, and soluble VEGFR-3, that normally maintain the cornea in an avascular state [10]. This leads to the formation of neovessels and neolymphatics, which further facilitate the trafficking of mature APCs to the cornea and to draining lymph nodes, where priming of naïve T cells or allosensitization occurs [8].

Both donor and recipient-derived APCs have the capacity to present alloantigen-MHC complexes to naïve T (Th0) cells. T cell stimulation through donor APCs or passenger leukocytes is known as the direct pathway of allorecognition, whereas the indirect pathway involves presentation of processed alloantigens to T cells through host APCs [11]. After activation, primed T cells undergo clonal expansion and give rise to CD4^+^IFN*γ*^+^T helper (Th1). Th1 cells are considered the main mediators of graft rejection and are the predominant cell population identified in the corneal stromal infiltrate of the grafts undergoing rejection [12, 13]. These effector CD4^+^ Th1 cells employ an array of mechanisms including the production of IL-2, IFN*γ*, and TNF-*α* cytokines, and FasL-mediated apoptosis of corneal endothelial cells to mount the delayed type hypersensitivity (DTH) immune response that results in the destruction of allogeneic corneal tissue [4, 11, 14, 15]. CD4^+^Foxp3^+^ regulatory T (Treg) cells, on the other hand, can interact with and regulate the function of both APCs and T cells and are a pivotal part of inducing immunologic tolerance against the graft [16]. The fate of the corneal allograft is highly dependent on the balance between the effector T cell and regulatory T cell responses, each deviating the immune response towards either rejection or tolerance.

## 3. Trafficking and Homing of Effector T Cells

### 3.1. Homing of Naïve T Cells to the Draining Lymph Nodes

**Figure 1:**
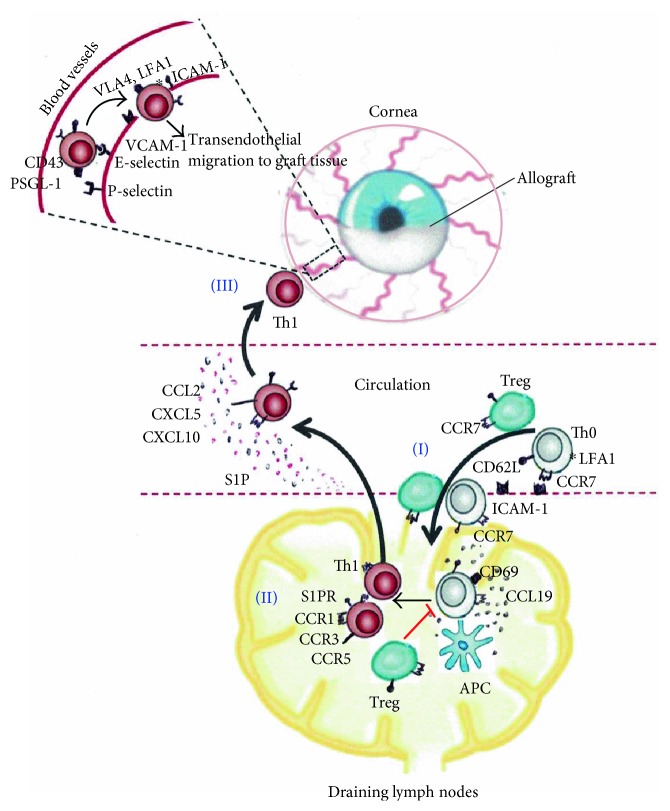
Overview of T cell trafficking in corneal allograft rejection. (I) Circulating naïve T (Th0) cells and regulatory T cells (Tregs) migrate to the parenchyma of draining lymph nodes via specialized postcapillary venules called high endothelial venules (HEVs). Interactions of T cell-expressed L-selectin (CD62L) and CC-chemokine receptor 7 (CCR7) with peripheral node addressin (PNAd) and CCL19 expressed on HEVs mediate the rolling and attachment of T cells to HEVs. The binding of lymphocyte function-associated antigen-1 (LFA-1) with intercellular adhesion molecule-1 (ICAM-1) initiates the transendothelial migration of T cells to the paracortical region of draining lymph nodes, where both naïve T cells and Tregs interact with antigen-presenting cells (APCs). CCR7-CCL19/CCL21 interactions are necessary for the motility of T cells within the draining lymph node to scan APCs for alloantigen presentation. T cell receptor activation, which occurs upon naïve T cell interaction with APCs, results in upregulation of CD69 by T cells. CD69 prevents the surface expression of sphingosine-1 phosphate receptor (S1PR) on T cells, rendering T cells desensitized to the S1P gradient, which in normal conditions drives T cells out of the draining lymph nodes. Activated T cells undergo clonal expansion and differentiate into effector CD4^+^ IFN*γ*^+^ Th1 cells. Tregs suppress allosensitization by inhibiting APC or T cell activation in the draining lymph nodes. (II) Upregulation of S1PR in conjunction with downregulation of CCR7 and L-selectin (CD62L) lymphoid homing molecules drive T cells out of the draining lymph node in response to high levels of S1P chemoattractant in the blood vessels. Antigen-primed Th1 cells express high levels of chemokine receptors, CCR1, CCR2, CCR3, and CCR5, which direct them to migrate towards the inflamed cornea in response to high chemokine gradient of peripheral tissue-specific chemokines including CCL2, CCL3, CCL4, CXCL10/IP-10, and CCL5/RANTES. (III) Th1 cells move within the blood vessels and migrate towards the corneal graft through interactions of E-selectin and P-selectin expressed by endothelial cell layer of vasculature with T cell-expressed ligands, glycosylated CD43, and PSGL-1. Arrest and subsequent transendothelial migration of effector Th1 cells towards the graft tissue are mediated through interactions of very late antigen-4 (VLA-4) and LFA-1 expressed on T cells with ICAM-1 and VCAM-1 selectins, which are highly expressed by endothelial cells of the blood vessels. This cascade of interactions collectively results in the migration of effector CD4^+^ IFN*γ*^+^ Th1 cells to the graft tissue, where they mount a delayed type hypersensitivity response against the allogeneic corneal graft.

Directed migration of T cells to the site of graft depends on a complex cascade of adhesion molecules, integrins, and chemokines. Circulating naïve T cells migrate to the parenchyma of draining lymph nodes through specialized postcapillary venules called high endothelial venules (HEVs) (Figure 1). Rolling of T cells in HEVs is mediated through the interaction of T cell-expressed L-selectin (CD62L) with peripheral node addressin (PNAd) expressed by HEVs [17]. Transendothelial migration of T cells to peripheral lymphoid tissues begins with firm attachment of T cells to HEVs through binding of CC-chemokine receptor 7 (CCR7) expressed on T cells with its ligands, CC-chemokine ligand 19 (CCL19) and CCL21, which are presented on HEVs [18]. CCR7 is a critical homing molecule for migration of both T cells and antigen-presenting cells to the secondary lymphoid tissues [18, 19]. The binding of CCR7 with CCL21 results in activation of the integrin lymphocyte function-associated antigen-1 (LFA-1), which mediates the arrest and transendothelial migration of T cells to the paracortical or T cell zones of the secondary lymph nodes through interactions with intercellular adhesion molecule-1 (ICAM-1) [20]. ICAM-1 is constitutively expressed on endothelial cells of limbal vessels and keratocytes in the cornea [21]. In corneal allografts undergoing rejection, the expression of ICAM-1 is increased on vascular endothelial cells and keratocytes, especially at the site of T cell infiltration [21]. Increased expression of ICAM-1 associated with increased leukocyte infiltration has also been demonstrated in a murine model of suture-induced corneal inflammation [22]. Once transmigrated to T cell zone of the lymphoid tissues, the mobility of T cells depends on the interactions of CCR7 with CCL19 and CCL21 ligands. T cell motility within the lymphoid tissue enhances T cell scanning of antigen-presenting cells and increases the possibility of T cell priming [23].

### 3.2. Egress of Effector T Cells from the Draining Lymph Nodes

The migration of primed T cells out of the lymph nodes rests on the sphingosine-1 phosphate (S1P) chemoattractant gradient and the expression levels of S1P receptor on T cells [24, 25]. S1P levels are significantly higher in blood vessels and lymphatics than in T cell zones of the secondary lymph nodes. This chemoattractant gradient directs T cells out of lymph nodes and ensures their recirculation [25]. However, during inflammation, T cells must remain in the draining lymph nodes for a sufficient amount of time to encounter antigen-bearing APCs. Initial T cell receptor activation and production of proinflammatory cytokines such as IFN*γ* result in upregulation of CD69 by T cells [17]. CD69 has been shown to internalize S1P receptor and prevent its surface expression on T cells; thereby desensitizing T cells to S1P gradient [24, 26, 27]. This results in retention of T cells in the secondary lymph nodes and increases their chances of encountering a mature antigen-bearing APC. Treatment with S1P type 1 receptor agonist has been shown to inhibit migration of lymphocytes and promote the survival of corneal allografts [28, 29]. After activation, T cell expression of S1P receptor increases, while the expression of lymphoid homing molecules L-selectin (CD62L) and CCR7 decreases substantially [30, 31]. These phenotypic changes lead to the migration of primed effector T cells out of the draining lymph nodes in response to the S1P gradient and prevent them from re-entering the lymphoid tissue (Figure 1()).

### 3.3. Migration of Effector T Cells to the Site of Graft

Several chemokines have been implicated in the recruitment of antigen-primed effector T cells towards the inflamed cornea (Figure 1()). Overexpression of CCL2, CCL3, CCL4, CXCL10/IP-10, and CCL5/RANTES chemokines has been detected in corneal allografts undergoing rejection [32]. Early production of CXCL1 in high-risk graft recipients upregulates the expression of T cell-specific chemokines, CXCL9 and CXCL10/IP-10, which recruit alloantigen-specific T cells to the site of graft [33]. Blockade of CXCL1 has been associated with decreased expression of CXCL9 and CXCL10, less graft infiltration of CD4^+^ T cells, and has been shown to significantly improve the survival of vascularized high-risk corneal allografts [33]. In addition to higher levels of selective chemokines, corneal graft rejection is also associated with upregulated expression of chemokine receptors CCR1, CCR2, and CCR5 [34]. CCR1 knockout corneal graft recipients demonstrate reduced T cell graft infiltration, suppressed expression of Th1-associated cytokines, and significantly improved corneal allograft survival [34].

Once migrated out of the lymphoid tissue towards the graft, effector T cells extravasate from the blood vessels to the graft tissue. Transmigration of T cells within blood vessels is mediated by selectins and integrins. Vascular endothelial cells express E- (endothelial-) and P- (platelet-) selectins [35]. Interactions of these selectins with their ligands, PSGL-1 and glycosylated CD43, which are expressed on circulating effector cells, initiate the transendothelial migration of effector Th1 cells [35, 36]. Selectins have been detected on the endothelium of vasculature in rejected corneal allografts [21]. It has been shown that the expression of both P-selectin and E-selectin by CD31^+^ vascular endothelial cells gets upregulated in rejected corneal allografts [37]. Both P- and E-selectins have been shown to mediate the recruitment of Th1 cells, but not Th2 cells in inflamed tissues [36]. Anti-E- and anti-P-selectin treatment decreases corneal infiltration of CD4^+^ T cells in transplanted mice, and E-selectin blockade significantly improves corneal allograft survival [37]. Firm attachment of effector T cells to vascular endothelial cells is mediated through interactions of T cell-expressed integrins, LFA-1 and very late antigen-4 (VLA-4) with ICAM-1 and VCAM-1, respectively. High expressions of ICAM-1 and its counter receptor LFA-1 have been detected in corneal allografts undergoing rejection [38]. Treatment of mice undergoing corneal transplantation with systemic anti-LFA-1 and anti-VLA-4 monoclonal antibodies significantly reduces effector Th1 responses and promotes corneal allograft survival [39, 40]. The expression of very late antigen-1 (VLA-1) integrin has also been demonstrated in grafted corneas [41]. VLA-1 is normally expressed on CD4^+^ T cells, and its expression is upregulated upon T cell activation [42]. Blockade of VLA-1 in a murine model of corneal transplantation has been shown to dramatically decrease infiltration of inflammatory cells, including T cells, and improve corneal allograft survival [41]. Interestingly, emerging data has shown that corneal allograft rejection is not only dependent on frequencies of infiltrating T cells but is also associated with a distinct phenotype of T cells that are highly mobile within the allograft stroma [43]. CCR5/CXCR3 signaling plays a significant role in the motility patterns of infiltrating T cells. Blockade of CCR5/CXCR3 pathway alters the phenotype of graft-infiltrating T cells to that of less mobile cells and significantly promotes corneal allograft survival [43].

## 4. Trafficking and Homing of Regulatory T Cells

Tregs modulate the immune response to the allograft by inducing and maintaining tolerance to alloantigens [44]. Tregs exert their regulatory function in two primary locations: the draining lymph nodes and the inflamed tissue [45, 46]. Tregs have been demonstrated to regulate the alloimmune response to corneal allograft primarily by suppressing T cell priming in the draining lymph nodes, rather than by controlling effector T cell responses in the inflamed cornea [47]. In addition, despite the increase in absolute frequencies of Tregs in the corneas undergoing rejection, the suppressive activity of graft-infiltrating Tregs is not significantly different between graft acceptors and rejectors. Lymph node-derived Tregs from graft acceptors, however, demonstrate significantly higher capacity in suppressing T cell activation [47, 48]. This highlights the significance of proper localization of Tregs to draining lymph nodes in the regulation of graft-directed immune response.

The majority of Tregs derived from draining lymph nodes of corneal allograft recipients express CCR7 and CD62L (Figure 1), and to a much lesser extent CD103 [48]. CCR7 and L-selectin (CD62L) homing receptors mediate the migration of Tregs from the thymus to the paracortical regions of draining lymph nodes through HEVs, where they make contact with antigen-presenting cells [49, 50]. CD103, on the other hand, has been implicated in retention of Tregs in inflamed tissues [51]. CCR7 appears to have a paradoxical function; it facilitates allosensitization by promoting the migration of antigen-presenting cells to draining lymph nodes, but at the same time, it enhances lymph node homing and suppressive function of Tregs [52, 53]. Data has shown that Tregs derived from draining lymph nodes of corneal allograft acceptors express higher levels of CCR7 compared to those of graft rejectors [48]. Ex vivo augmentation of CCR7 expression by naïve Tregs through CCL21 stimulation promotes Treg homing to draining lymph nodes and enhances corneal allograft survival [48].

## 5. Conclusions

The migration of leukocytes to the site of inflammation is a complex and multistep process, which is indispensable to induction of optimal host immune response. This directed trafficking forms the basis of effector immune responses that ultimately lead to destruction of the allograft tissue. The intricate interactions between chemokines and adhesion molecules and their counterpart receptors on immune cells have been intriguing research targets in various transplantation contexts. A better understanding of the key molecular players involved in organ-specific targeted migration of leukocytes, and their effector function at the site of graft could provide therapeutic opportunities to manipulate these interactions in favor of prolonging corneal allograft survival.
